# Trends in Suicidality and Bullying among New York City Adolescents across Race and Sexual Identity: 2009–2019

**DOI:** 10.1007/s11524-024-00860-0

**Published:** 2024-05-10

**Authors:** Devin English, Elizabeth Kelman, Nneka Lundy De La Cruz, Azure B. Thompson, Karolyn Le, Marné Garretson, Aishwarya L. Viswanath, Diksha Brahmbhatt, Cynthia Lockwood, Danielle R. Busby, Marivel Davila

**Affiliations:** 1 Rutgers School of Public Health, 1 Riverfront Plaza, Newark, NJ 07102 USA; 2 New York City Department of Health and Mental Hygiene, 42-09 28th Street, Long Island City, NY 11101 USA; 3 SUNY Downstate Health Sciences University, 450 Clarkson Avenue, Brooklyn, NY 11203 USA; 4 University of Texas Medical Branch, 301 University Boulevard, Galveston, TX 77555 USA

**Keywords:** Sexual and Gender minorities, Black or African American, Adolescent, Students, Suicidal ideation, Suicide attempt, Bullying, Cyberbullying

## Abstract

Despite evidence showing rising suicidality among lesbian, gay, and bisexual (LGB) and Black adolescents, separately, there is scant research on suicide risk trajectories among youth groups across both racial and sexual identities. Thus, we examined trajectories of self-reported suicidal ideation and attempt and their associations with bullying among New York City-based adolescents. We analyzed 2009–2019 NYC Youth Risk Behavior Survey data. We ran weighted descriptive and logistic regression analyses to test for trends in dichotomous suicidal ideation, suicide attempt, bullying at school, and e-bullying variables among students across both race/ethnicity and sexual identity. We assessed associations between suicidality trends and bullying with logistic regressions. Models controlled for age and sex. Suicidal ideation and attempt were 2 and 5 times more likely among LGB than heterosexual participants, respectively. Bullying at school and e-bullying were 2 times more likely among LGB than heterosexual participants. Black LGB participants were the only LGB group for which both suicidal ideation (AOR = 1.04, SE = .003, *p* < .001) and attempt (AOR = 1.04, SE = .004, *p* < .001) increased over time. Both increased at accelerating rates. Conversely, White LGB participants were the only LGB group for which both suicidal ideation (AOR = 0.98, SE = .006, *p* < .001) and attempt (AOR = 0.92, SE = .008, *p* < .001) decreased over time. These changes occurred in parallel with significant bullying increases for Black and Latina/o/x LGB adolescents and significant bullying decreases for White LGB adolescents. Bullying was positively associated with suicidal ideation and attempt for all adolescents. Findings suggest resources aimed at curbing rising adolescent suicide should be focused on Black LGB youth.

**Supplementary Information:**

The online version contains supplementary material available at 10.1007/s11524-024-00860-0.

## Introduction

Although lesbian, gay, bisexual (LGB), and other sexual minority youth aged 13–17 years comprise 9% of the total U.S. population [1], they account for 36% of suicide attempts, a rate over three times higher than heterosexual adolescents [2]. From 2009 to 2017, LGB youth accounted for an increasing proportion of suicide attempts over time [2]. Over this same period, Black adolescents were the only adolescent racial group for which suicide attempts increased [3]. Despite these indicators of rising suicidality among LGB and Black adolescents, there is scant research on suicide risk trajectories among youth groups across both racial and sexual identities over this period. However, research does suggest Black and Latina/o/x LGB adolescents may experience disproportionate suicidality due to bullying [4]. Thus, the present study examined trajectories of suicidal ideation and attempt among New York City high school students across racial identity and sexual identity from 2009 to 2019, and whether these trajectories were associated with bullying.

Studies consistently find vast suicide inequities between LGB youth and their heterosexual counterparts [5–9]. Data from the Youth Risk Behavior Surveillance System (YRBSS) [9, 10] indicate LGB high school students are 2.9 times more likely to have suicidal ideation and 4.6 times more likely to attempt suicide than their heterosexual peers [11]. Analysis of suicide trends suggests these inequities have worsened over recent years [2]. Although early suicidality research suggested that White communities may be at higher risk than other racial/ethnic communities [12], more recent studies indicate youth suicide rates are the highest, and increasing, among Black children [13–16]. Moreover, research examining suicide trends among adolescents found that although suicidal ideation decreased for all racial/ethnic groups, attempts increased only among Black adolescents [3]. This is critical since research suggests Black and Latina/o/x LGB adults may be at higher risk for suicide attempt than their White peers [17], and Black and Latina/o/x bisexual boys and Black lesbian girls may be at higher risk of suicidal ideation compared to their White peers [18]. However, we are unaware of any research examining recent trends in suicidal ideation, suicide attempt, and bullying among adolescents across both racial and sexual identity groups (e.g., Black heterosexual, Black LGB, Latina/o/x heterosexual, Latina/o/x LGB).

Studies conceptualizing drivers of increasing suicide rates among Black and LGB adolescents have posited that Black LGB youth may be at high risk for suicidal ideation and attempt because they face racist and heterosexist bullying, among other forms of oppression [19, 20]. This is consistent with interpersonal theories that suggest perceived burdensomeness and thwarted belongingness linked to interpersonal rejection are critical contributors to suicide risk [20–22]. Public health applications of intersectionality frameworks posit that health inequities at the intersection of racial and sexual minority identities reflect the effects of interlocking systems of oppression (e.g., racism, heterosexism) [23–27]. Thus, documentation of suicide rates across racial and sexual identity, and the examination of bullying as a manifestation of the intersecting oppression driving those rates, is necessary to identify and eliminate suicide inequities.

Evidence shows bullying predicts suicide risk among adolescents [18, 28–31]. Bullying in schools and on the internet (i.e., e-bullying) is 1.5 to 2.24 times more likely to target LGB adolescents than their heterosexual peers [30, 32–34]. Additionally, suicide attempt rates among Black and Latina/o/x LGB late adolescents and young adults may be inequitably increased by heterosexist discrimination [4]. However, other research with YRBSS data does not show differences in the effects of bullying across race and ethnicity among LGB youth [18]. Thus, it is important to examine bullying as a form of discrimination that may be contributing to recent increases in adolescent suicide inequities across race and sexual identity.

To assess recent suicidality trends across racial and sexual identity groups and test bullying as a potential contributor to these trends, we examined trajectories of suicidal ideation and attempt across racial and sexual identity groups and their associations with school-based bullying and e-bullying among NYC public and charter high school students. We used the Youth Risk Behavior Survey (YRBS), which has shown rates of suicide attempt and bullying that are comparable to national estimates [30]. We focused on years 2009 through 2019 because measurement of e-bullying started in 2009 and the 2021 data were biased by COVID-19-related school-based data collection limitations. Since our examination of suicidal ideation and suicide attempt trends was descriptive, we did not test specific hypotheses for each subgroup. However, given recent evidence that suicide attempt rates are increasing among LGB [2] and Black [3] youth, we expected that Black LGB adolescents would show significant increases in suicide attempt across the study period. Given the wealth of evidence linking bullying to suicidal ideation and attempts [18, 28–31], we expected bullying to be positively associated with ideation and attempt over this period for all groups.

## Methods

We analyzed data from the NYC YRBS. As part of the Centers for Disease Control and Prevention’s nationwide YRBSS, the NYC YRBS is a biennial, school-based survey conducted by the NYC Department of Health and Mental Hygiene in collaboration with the NYC Department of Education. As a self-administered, anonymous survey, it employs a stratified, two-stage, cluster design to produce a representative sample of students in grades 9–12 attending NYC public, charter, or vocational schools. In the first stage, schools are randomly sampled with probability proportional to schools’ enrollment sizes. In the second stage, classrooms are randomly sampled, excluding English as a Second Language and special education classes. The survey includes questions specific to six areas of health-related risk behaviors that contribute to leading causes of mortality and morbidity. Our study includes data from a weighted sample of 228,626 participants across six surveys conducted from 2009 to 2019.

### Measures

The supplemental appendix includes the questions and response options for our study variables. We used students’ self-reported race and ethnicity. We combined students who self-identified as Hispanic/Latino into one category. We use the terminology “Latina/o/x” for participants who responded yes to this question and to acknowledge that we do not know participants’ gender identity since the YRBS only asked about sex assigned at birth [35]. We did not examine data for American Indian/Alaska Native (*n*_LGB_ = 98), Native Hawaiian/Other Pacific Islander (*n*_LGB_ = 98), or Multiple Non-Hispanic/Latino (*n*_LGB_ = 219) youth because sample sizes for LGB participants were too small to derive reliable estimates. We dichotomized sexual identity into heterosexual or LGB to ensure reliable estimates. Students who responded “Not Sure” (6.1%) were not included in the analysis since it was unclear whether their response referred to questioning their sexual orientation or not understanding the question. Bullying variables included being bullied on school property as well as internet-based bullying (e-bullying), which was first assessed in 2009. Consistent with past studies examining suicide attempts among YRBS participants [3], and to be consistent with the suicidal ideation item, we dichotomized the attempt variable.

### Analysis Plan

We ran descriptive and logistic regression analyses using SPSSv27 to test for trends in suicidal ideation, suicide attempt, bullying at school, and e-bullying variables among participants disaggregated by race/ethnicity and sexual identity. Consistent with the National Center for Health Statistics Guidelines [36] and past studies of suicidality trends [3], we ran logistic regressions on stacked individual-level data from 2009 to 2019. For suicidality and bullying logistic regressions, we entered the centered year and squared centered year as independent variables to assess linear and quadratic growth, respectively. We ran a second set of logistic regressions, where we entered mean bullying at school and e-bullying as predictors of suicidal ideation and attempt. All models controlled for age and sex assigned at birth. The results are weighted using YRBS population weights so that inferences apply to regular public, charter, and vocational school students in grades 9–12.

## Results

Table 1 includes demographics stratified by sexual identity across study variables. LGB participants were 11% of the sample. Among LGB participants, 74% identified as bisexual. Most (73%) LGB participants were assigned female sex at birth. LGB participants tended to be older and in higher grades compared to heterosexual participants. The majority of participants were Black or Latina/o/x in both groups. There was a greater proportion of White and Asian participants in the heterosexual than LGB group.

**Table 1:** Demographic information by sexual identity

	Overall total (weighted *n* = 262,461)			Sample by sexual identity					
				Heterosexual (weighted *n* = 203,427)			LGB (weighted *n* = 25,199)		
	*Unweighted n*	*Weighted n*	*Weighted* %	*Unweighted n*	*Weighted n*	*Weighted%*	*Unweighted n*	*Weighted n*	*Weighted%*
Sexual identity									
Heterosexual	47,409	203,000	89.0	–	–	–	–	–	–
Lesbian/Gay	1565	7000	2.9	–	–	–	1565	7000	26.0
Bisexual	4556	19,000	8.2	–	–	–	4556	19,000	74.0
Sex assigned at birth							*χ*2(1) = 494.62, *p* < 0.001		
Female	31,654	129,000	49.7	23,534	96,000	47.1	4459	18,000	72.7
Male	29,042	131,000	50.3	23,711	107,000	52.9	1554	7000	27.3
Grade							*χ*^2^(4) = 14.13, *p* = 0.003		
9th grade	15,769	76,000	29.4	11,995	58,000	28.8	1499	7000	27.6
10th grade	15,968	70,000	26.9	12,339	54,000	26.9	1662	7000	27.5
11th grade	14,525	58,000	22.2	11,505	46,000	22.7	1402	5000	21.6
12th grade	14,004	55,000	21.0	11,026	43,000	21.3	1433	6000	22.4
Age							*χ*^2^(6) = 4.11, *p* < 0.001		
12 years old or younger	280	1000	0.5	142	< 1000	0.3	65	< 1000	1.1
13 years old	1618	8000	3.0	1202	6000	2.9	171	1000	2.9
14 years old	12,706	61,000	23.3	9866	47,000	23.2	1159	5000	21.5
15 years old	15,495	69,000	26.2	12,102	54,000	26.4	1554	6000	25.9
16 years old	14,965	60,000	23.1	11,735	47,000	23.3	1514	6000	23.4
17 years old	12,763	50,000	19.2	10,023	39,000	19.4	1280	5000	19.9
18 years old or older	3097	12,000	4.8	2216	9000	4.4	356	1000	5.4
Race/ethnicity							*χ*^2^(3) = 48.35, *p* < 0.001		
Asian	6571	39,000	15.9	5480	32,000	16.8	355	2000	9.2
Black or African American	15,121	77,000	31.5	11,585	59,000	30.8	1454	7000	31.7
White	7218	35,000	14.3	6121	29,000	15.4	538	3000	11.2
Latina/o/x	25,927	93,000	38.3	19,739	70,000	37.0	3098	11,000	47.9

Female and male categories include cisgender and transgender female and male participants

Weighted *N* rounded to the nearest thousands

**Fig. 1:**
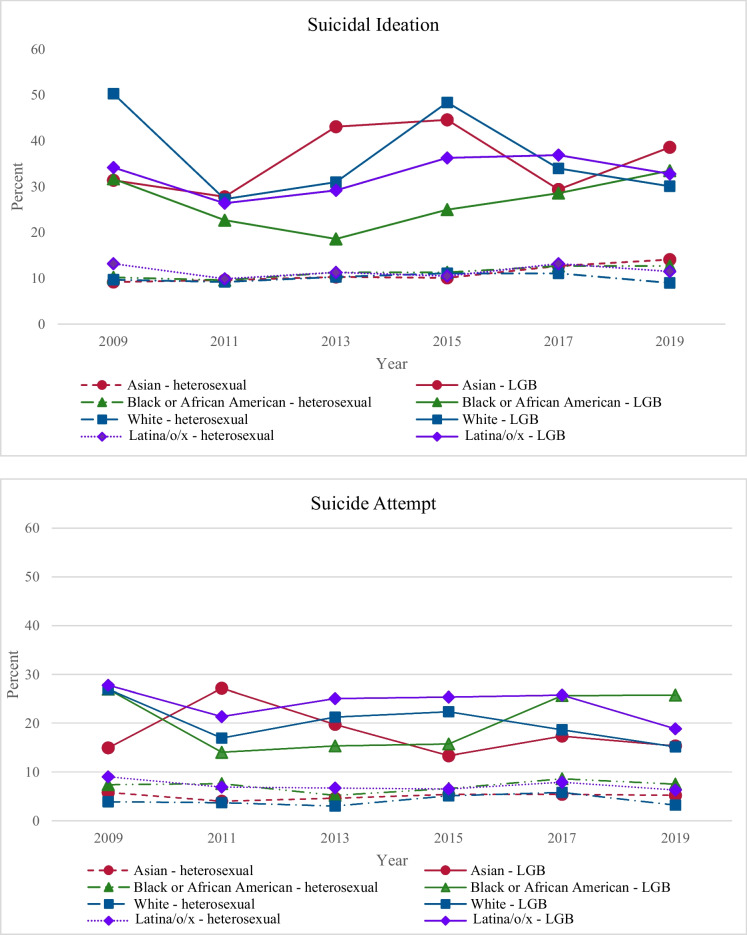
Yearly suicidal ideation and attempt means by race/ethnicity and sexual identity

Figure 1 shows the trends in past-year suicidal ideation and attempt from 2009 to 2019 across race/ethnicity and sexual identity. During this period, the weighted overall prevalence of suicidal ideation and attempt among LGB participants was 22% and 32%, respectively, and 11% and 6% among heterosexual participants, respectively. Adjusted Odds Ratios (AOR) for suicidal ideation and attempt across race and ethnicity and sexual identity are included in Table 2.

**Table 2:** Logistic regression testing for trends in suicide ideation and attempt

	Suicide ideation				Suicide attempt			
	*b*	*SE*	*AOR*	*p-value*	*b*	*SE*	*AOR*	*p-value*
LGBQ								
Black LGBQ								
Linear	0.037	0.003	1.04	** < 0.001**	0.041	0.004	1.04	** < 0.001**
Quadratic	0.024	0.001	1.02	** < 0.001**	0.025	0.001	1.03	** < 0.001**
Asian LGBQ								
Linear	0.000	0.007	1.00	0.992	− 0.044	0.009	0.96	** < 0.001**
Quadratic	− 0.009	0.002	0.99	** < 0.001**	− 0.001	0.003	1.00	0.618
Latina/o/x LGBQ								
Linear	0.020	0.003	1.02	** < 0.001**	− 0.036	0.003	0.97	** < 0.001**
Quadratic	− 0.001	0.001	1.00	0.526	− 0.008	0.001	0.99	** < 0.001**
White LGBQ								
Linear	− 0.021	0.006	0.98	** < 0.001**	− 0.086	0.008	0.92	** < 0.001**
Quadratic	0.005	0.002	1.01	** < 0.05**	− 0.008	0.002	0.99	** < 0.001**
Heterosexual								
Black heterosexual								
Linear	0.034	0.002	1.04	** < 0.001**	0.010	0.002	1.01	** < 0.001**
Quadratic	0.000	0.001	1.00	0.373	0.007	0.001	1.01	** < 0.001**
Asian heterosexual								
Linear	0.053	0.002	1.05	** < 0.001**	0.011	0.003	1.01	** < 0.001**
Quadratic	0.005	0.001	1.01	** < 0.001**	0.005	0.001	1.01	** < 0.001**
Latina/o/x heterosexual								
Linear	0.007	0.001	1.01	** < 0.001**	− 0.018	0.002	0.98	** < 0.001**
Quadratic	0.005	0.000	1.01	** < 0.001**	0.004	0.001	1.01	** < 0.001**
White heterosexual								
Linear	0.008	0.002	1.01	** < 0.01**	0.021	0.004	1.02	** < 0.001**
Quadratic	− 0.007	0.001	0.99	** < 0.001**	− 0.005	0.001	1.00	** < 0.001**

Adjusted Odds Ratios (AORs) pertain to the 10-year study period

The significant *p*-values are bolded

For suicidal ideation among LGB participants, Black and Latina/o/x LGB participants showed significant linear increases, with a quadratic increase and nonsignificant quadratic change, respectively. White LGB participants showed a significant linear decrease with a quadratic increase. Asian LGB participants showed a nonsignificant linear change. For suicide attempts among LGB participants, all racial/ethnic groups showed a significant linear decrease except for Black LGB participants, for whom there was a linear and quadratic increase. Latina/o/x and White LGB participants showed quadratic decreases. 

For suicidal ideation among heterosexual participants, all ethnic/racial heterosexual groups showed linear increases, though linear increases among Black and Asian heterosexual participants were 4–5 times greater than those for White and Latina/o/x heterosexual participants. Increases among Asian and Latina/o/x heterosexual participants showed a quadratic increase, while those among White heterosexual participants showed a quadratic decrease. For suicide attempts among heterosexual participants, all racial/ethnic heterosexual groups showed significant linear increases except for Latina/o/x heterosexual participants, for whom there was a decrease. All heterosexual groups showed significant quadratic increases except for White heterosexual participants, for whom there was a quadratic decrease.

**Fig. 2:**
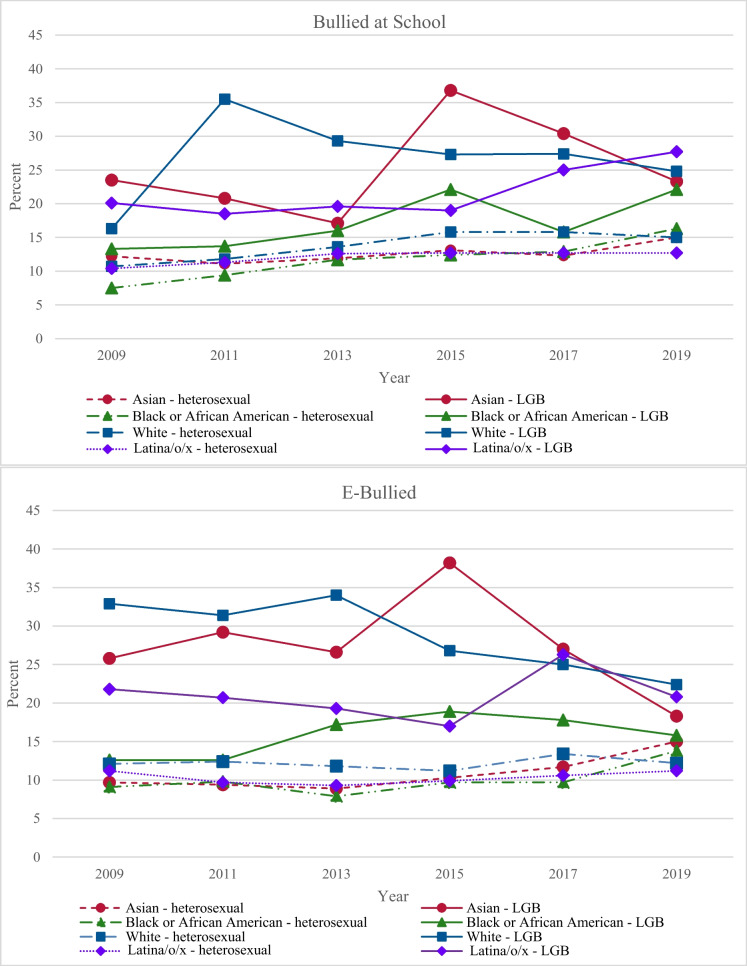
Yearly bullying at school and e-bullying means by race/ethnicity and sexual identity

The weighted overall prevalence for bullying at school and e-bullying among LGB participants was 22% and 21%, respectively, and 12.5% and 10.7% among heterosexual participants, respectively. The disaggregated mean trends for bullying at school and e-bullying are depicted in Fig. 2.

For bullying at school among LGB participants, Black (AOR = 1.06, SE = 0.004, *p* < 0.001) and Latina/o/x (AOR = 1.01, SE = 0.001, *p* < 0.001) LGB participants showed significant linear increases with a quadratic increase (AOR = 0.996, SE = 0.00, *p* = 0.007) and decrease (AOR = 1.01, SE = 0.001, *p* < 0.001), respectively. White LGB participants showed a significant linear decrease (AOR = 0.98, SE = 0.007, *p* < 0.001) with a quadratic decrease (AOR = 0.99, SE = 0.001, *p* = 0.001). Asian LGB participants showed a nonsignificant linear change (AOR = 1.00, SE = 0.008, *p* = 0.78). For e-bullying among LGB participants, Black (AOR = 1.04, SE = 0.004, *p* < 0.001) and Latina/o/x (AOR = 1.01, SE = 0.003, *p* < 0.001) LGB participants showed significant linear increases with a quadratic increase (AOR = 0.99, SE = 0.001, *p* < 0.001) and decrease (AOR = 1.01, SE = 0.001, *p* < 0.001), respectively. White LGB participants (AOR = 0.94, SE = 0.007, *p* < 0.001) and Asian LGB participants (AOR = 0.91, SE = 0.008, *p* < 0.001) showed significant linear decreases with a nonsignificant quadratic change (AOR = 1.01, SE = 0.003, *p* = 0.08) and a quadratic decrease (AOR = 0.97, SE = 0.002, *p* < 0.001), respectively.

For bullying at school among heterosexual participants, Black (AOR = 1.08, SE = 0.002, *p* < 0.001), Latina/o/x (AOR = 1.02, SE = 0.001, *p* < 0.001), and White (AOR = 1.05, SE = 0.002, *p* < 0.001) heterosexual participants showed significant linear increases. Black (AOR = 0.997, SE = 0.001, *p* < 0.001), Latina/o/x (AOR = 0.995, SE = 0.001, *p* < 0.001), and White (AOR = 0.993, SE = 0.001, *p* < 0.001) heterosexual participants also showed significant quadratic decreases. Asian heterosexual participants showed linear (AOR = 1.03, SE = 0.002, *p* < 0.001) and quadratic (AOR = 1.01, SE = 0.001, *p* < 0.001) increases. For e-bullying among heterosexual participants, Black (AOR = 1.04, SE = 0.002, *p* < 0.001), Latina/o/x (AOR = 1.01, SE = 0.001, *p* < 0.001), White (AOR = 1.01, SE = 0.002, *p* < 0.001), and Asian (AOR = 1.06, SE = 0.002, *p* < 0.001) heterosexual participants showed significant linear increases. Black (AOR = 1.01, SE = 0.001, *p* < 0.001), Latina/o/x (AOR = 1.01, SE = 0.001, *p* < 0.001), and Asian (AOR = 1.01, SE = 0.001, *p* < 0.001) heterosexual participants showed quadratic increases, but White (AOR = 0.998, SE = 0.002, *p* = 0.23) heterosexual participants showed a nonsignificant quadratic change. For the models that examined associations between suicidal ideation and attempt and bullying at school and e-bullying, both forms of bullying were positively associated with suicidal ideation and attempt across all groups (Table 3).

**Table 3:** Logistic regression testing for associations between suicidality and bullying

	Suicidal ideation				Suicidal attempt			
	*b*	*SE*	*AOR*	*p-value*	*b*	*SE*	*AOR*	*p-value*
LGB								
Black LGB								
E-bullied	0.373	0.032	1.45	** < 0.001**	0.910	0.037	2.48	** < 0.001**
Bullied	0.932	0.030	2.54	** < 0.001**	0.763	0.036	2.15	** < 0.001**
Asian LGB								
E-bullied	0.558	0.049	1.75	** < 0.001**	1.056	0.063	2.87	** < 0.001**
Bullied	0.386	0.050	1.47	** < 0.001**	0.158	0.067	1.17	**0.018**
Latinx LGB								
E-bullied	0.603	0.023	1.83	** < 0.001**	0.297	0.028	1.35	** < 0.001**
Bullied	0.858	0.022	2.36	**0.000**	0.944	0.027	2.57	** < 0.001**
White LGB								
E-bullied	1.014	0.045	2.76	** < 0.001**	0.946	0.054	2.58	** < 0.001**
Bullied	0.763	0.046	2.15	** < 0.001**	0.874	0.056	2.40	** < 0.001**
Heterosexual								
Black heterosexual								
E-bullied	0.887	0.016	2.43	**0.000**	0.606	0.023	1.83	** < 0.001**
Bullied	0.873	0.015	2.39	**0.000**	0.590	0.022	1.81	** < 0.001**
Asian heterosexual								
E-bullied	0.850	0.022	2.34	** < 0.001**	1.018	0.031	2.77	** < 0.001**
Bullied	0.653	0.021	1.92	** < 0.001**	0.390	0.033	1.48	** < 0.001**
Latinx heterosexual								
E-bullied	1.141	0.014	3.13	**0.000**	0.955	0.020	2.60	**0.000**
Bullied	0.836	0.014	2.31	**0.000**	0.584	0.019	1.79	** < 0.001**
White heterosexual								
E-bullied	1.075	0.021	2.93	**0.000**	0.884	0.034	2.42	** < 0.001**
Bullied	0.991	0.021	2.69	**0.000**	0.635	0.034	1.89	** < 0.001**

Adjusted Odds Ratios (AORs) pertain to the 10-year study period

The significant *p*-values are bolded

## Discussion

This study examined suicidal ideation, suicide attempt, and bullying trajectories across racial and sexual identity groups among a NYC YRBS sample from 2009 to 2019. Among LGB students, for whom suicidal ideation and attempt were twice and five times more likely than for heterosexual students, respectively, Black LGB participants were the only group for which both suicidal ideation and attempt increased. Bullying at school and e-bullying were each about two times more likely among LGB than heterosexual students, and Black LGB participants were the only LGB group for which both forms of bullying were increasing at increasing rates. For all students, we found that bullying was positively associated with suicidal ideation and attempt across time. These results suggest that research and intervention resources should prioritize identifying and rectifying drivers of suicide inequities for Black LGB adolescents, including preventing and combatting the negative impacts of bullying.

The findings that LGB students were twice as likely to experience suicidal ideation and five times more likely to attempt suicide were consistent with past studies showing vast suicide inequities between LGB and heterosexual adolescents [5–8, 11]. The fact that Black LGB students were the only LGB group for which suicidal ideation and attempt increased during 2009 to 2019 extends past research that has shown recent increases in suicide attempts among LGB [2] and Black communities [3, 13–15], separately. This result supports research that suggests that Black LGB adolescents, who face oppressive conditions at the intersection of racism and heterosexism [23], may be at heightened risk for suicidality compared to their heterosexual and non-Black peers [37]. As such, efforts to curb rising rates of suicidality among LGB [38] and Black [39, 40] adolescents will likely be most effective by focusing on supporting and protecting Black LGB adolescents.

We also found Latina/o/x LGB students showed a significant increase in suicidal ideation across the study period. This is consistent with research indicating that suicidality may be increasing among Latina adolescents [41] and indicates that Latina/o/x LGB youth may be at inequitable risk for these increases. Additionally, we found that White LGB participants showed decreased suicidal ideation and attempt across time. While White LGB participants experienced higher rates of suicidal ideation and attempt than all groups of heterosexual participants, their suicidality rates declined compared to their Black, Latina/o/x, and Asian LGB peers. In fact, despite White LGB participants having among the highest levels of suicidal ideation and attempt in 2009, they were the lowest LGB group in both outcomes by 2019.

These changes in LGB suicidal ideation and attempt were linked to bullying. Bullying in school and e-bullying both significantly increased among Black and Latina/o/x LGB youth while decreasing among White LGB adolescents. Bullying is one way in which systems of oppression manifest: targets of bullying are often individuals with marginalized social status, including racial and sexual minorities [42]. Our results show bullying in school and e-bullying were significantly associated with suicidal ideation and attempt across time for each of these groups. As such, increases in bullying may be partially driving increases in Black and Latina/o/x LGB suicidality, while decreases in bullying may be partially driving decreases in White LGB suicidality. Additional research should consider how whiteness, including resources, opportunities, and support allocated to White LGB youth, may serve as a protective factor in suicidality for White LGB adolescents [43].

While there was no significant change in suicidal ideation or bullying at school over the study period for Asian LGB participants, both indicators peaked in 2015, and Asian LGB participants had the highest mean levels of suicidal ideation in 2019. Similarly, while e-bullying reported by Asian LGB participants significantly decreased across the study period, there was a substantial spike in e-bullying from 2013 to 2015. Additional exploration and consideration of these trends among Asian LGB participants should be pursued in future research, particularly in the wake of COVID-19-related spikes in anti-Asian hate crimes [44].

Among heterosexual participants, for whom the prevalence of suicidality and bullying were consistently below those of LGB participants, there were still notable trends. Suicidal ideation increased among all groups, though the increases for Black and Asian heterosexual participants were four and five times higher than those of other groups, respectively. Latina/o/x participants were the only heterosexual group for which suicide attempts decreased over the study period. Additional inquiry should investigate the trends among both Latina/o/x LGB and heterosexual youth showing concurrent increases in suicidal ideation and decreases in attempts. Bullying at school and e-bullying increased among all heterosexual groups, though increases in bullying at school were highest for Black heterosexual participants. Increases in e-bullying were highest for Asian heterosexual participants, the only heterosexual group for which increases in both forms of bullying were accelerating across the study period.

Our results support screening for bullying and suicidality as a critical pediatric mental health competency [45], particularly for LGB adolescents of color. Our findings also support past recommendations for healthcare providers to advocate for evidence-based school and community anti-bullying programs as part of their practice [46]. This should include supporting state-level structural interventions to prevent bullying [47].

While there are several strengths to this analysis of YRBS suicidality and bullying rates and associations among adolescents disaggregated by racial/ethnic and sexual identity groups, there are also several limitations worth noting. First, this study used NYC YRBS data, and while these data have been relatively consistent with national YRBS estimates for suicidality and bullying [30], these results, nonetheless, may not be generalizable outside of NYC or other large cities’ public and charter high school students. Additionally, given limitations with YRBS data collection around race/ethnicity and gender identity, we were unable to examine suicidality and bullying trajectories among multiracial/ethnic, indigenous, or Black Latina/o/x participants, nor among trans and gender expansive adolescents. Future research using national YRBS datasets that illuminate trends in suicidality and bullying among these groups, as well as break down results across gender and sexual identity (e.g., bisexual alone, lesbian alone) [18], will be essential. The YRBS bullying variables were also relatively blunt and non-specific given they used a dichotomous item to assess whether any form of bullying occurred over the past year. Without information on the identity targets and frequency information, we are unable to conclude that any increases in suicidality across racial/ethnic and sexual minority identities were associated with racism and heterosexism, and we have no information about how frequently these experiences were occurring. Given the serial cross-sectional nature of the YRBS data collection, we were not able to examine longitudinal, within-person trends, and associations between suicidality or bullying. Future research should consider examining parallel process models of longitudinal suicidal ideation and attempt and oppression variables. Finally, given these data were collected prior to the COVID-19 pandemic, and some research indicates that suicide may be inequitably increasing among Black youth since its onset [48], it will be critical that future studies examine post-COVID suicidality across racial and sexual identity.

## Conclusion

Our results suggest that increases in suicide attempts among Black youth may be inequitably pronounced among Black LGB adolescents and linked to increases in bullying at school and e-bullying. Analysis of a representative sample of NYC public high school students across 2009–2019 showed that LGB participants were two times more likely to experience suicidal ideation, over five times more likely to attempt suicide, and about two times as likely to experience bullying as their heterosexual peers. Among LGB adolescents, Black LGB youth were the only group for whom suicidal ideation and attempt were both increasing, rates that were linked to increases in both forms of bullying. Overall, our findings indicate that calls to address increasing suicide among Black youth [39, 40] should focus research and intervention resources on averting suicidality among Black LGB youth.

### Supplementary Information

Below is the link to the electronic supplementary material.

Supplementary file1 (DOCX 15 KB)

## Data Availability

Data and study materials from this study are available from the New York City Department of Health and Mental Hygiene on reasonable request. Study analysis code is available from the corresponding author on reasonable request.
