# Supplementary material for: Trends in Suicidality and Bullying among New York City Adolescents across Race and Sexual Identity: 2009–2019
Source: J Urban Health. 2024 May 10;101(3):451–63. doi: 10.1007/s11524-024-00860-0 (PMC11189860; doi:10.1007/s11524-024-00860-0)
Supplement: Supplementary file 1 — Supplementary file1 (DOCX 15 KB) [file 11524_2024_860_MOESM1_ESM.docx]

Table 1. Youth Risk Behavior Survey Questions, New York City, 2009-2019

| *Demographics* | | |
| --- | --- | --- |
| Survey question | Wording of survey question | Response options |
| Sex | What is your sex? | A. Female  B. Male |
| Hispanic or Latino | Are you Hispanic or Latino? | A. Yes  B. No |
| Race | What is your race?  (Select one or more responses.) | A. American Indian or Alaska Native  B. Asian  C. Black or African American  D. Native Hawaiian or Other Pacific Islander  E. White |
| *Sexual identity* | | |
| **Survey question** | **Wording of survey question** | **Response options** |
| Sexual identity | Which of the following best describes you? | A. Heterosexual (straight)  B. Gay or lesbian  C. Bisexual  D. Not sure |
| *Bullying* | | |
| Introduction: “Bullying is when 1 or more students tease, threaten, spread rumors about, hit, shove, or hurt another student over and over again. It is not bullying when 2 students of the same strength or power argue or fight or tease each other in a friendly way.” | | |
| **Survey question** | **Wording of survey question** | **Response options** |
| Bullied at school | During the past 12 months, have you ever been bullied on school property? | A. Yes  B. No |
| Electronically bullied | During the past 12 months, have you ever been electronically bullied? (Count being bullied through texting, Instagram, Facebook, or other social media.) | A. Yes  B. No |
| *Suicidal Behaviors* | | |
| Introduction: “The next 4 questions ask about sad feelings and attempted suicide. Sometimes people feel so depressed about the future that they may consider attempting suicide, that is, taking some action to end their own life.” | | |
| **Survey question** | **Wording of survey question** | **Response options** |
| Suicidal ideation | During the past 12 months, did you ever seriously consider attempting suicide? | A. Yes  B. No |
| Suicide attempt | During the past 12 months, how many times did you actually attempt suicide? | A. 0 times  B. 1 time  C. 2 or 3 times  D. 4 or 5 times  E. 6 or more times |
